# Catching a (Double-Strand) Break: The Rad51 and Dmc1 Strand Exchange Proteins Can Co-occupy Both Ends of a Meiotic DNA Double-Strand Break

**DOI:** 10.1371/journal.pgen.1005741

**Published:** 2015-12-31

**Authors:** Amy J. MacQueen

**Affiliations:** Department of Molecular Biology and Biochemistry, Wesleyan University, Middletown, Connecticut, United States of America; National Cancer Institute, UNITED STATES

Broken DNA can be repaired by homologous recombination mechanisms, which initially align both ends of a DNA double-strand break (DSB) with a homologous repair template. Particularly in the context of a crowded eukaryotic nucleus, it remains mysterious how the two ends of a broken DNA molecule coordinate their actions to identify an appropriate template and initiate repair. In this issue, Brown et al. [1] provide new information that bears on how this is accomplished during meiosis.

Homologous recombination is employed on a grand scale in germ cells undergoing meiosis in order to facilitate a nucleus-wide homology search that will ultimately establish links between previously unassociated homologous chromosomes [2,3]. During meiosis, homologous recombination initiates with programmed DSBs; the regulated repair of such meiotic DSBs leads to the formation of crossover recombination events between homologous chromosomes. Crossovers, in conjunction with sister chromatid cohesion, provide the attachments between homologous chromosomes that ensure their proper disjunction on the meiotic spindle. The meiotic nucleus thus provides a powerful system for investigating the molecular features and dynamics of early recombination intermediates in the context of the eukaryotic nucleus. The central task of meiosis also poses an interesting challenge to recombination machinery, as its aim is to reinforce interactions between relatively distant homologous chromatids rather than spatially proximal sister chromatids. Notably, both ends of a single broken DNA molecule must identify the same distant repair template but behave differently with respect to one another at the site of repair; the identification of single-end invasion (SEI) meiotic recombination intermediates in budding yeast [4] suggests that the ends of meiotic DSBs engage with a homologous template in a sequential fashion, as postulated in classic double-strand break repair (DSBR) models [5]. These challenges raise the question: How are opposite ends of a DSB controlled such that they coordinately interface with the same homologous target DNA?

**Fig 1:**
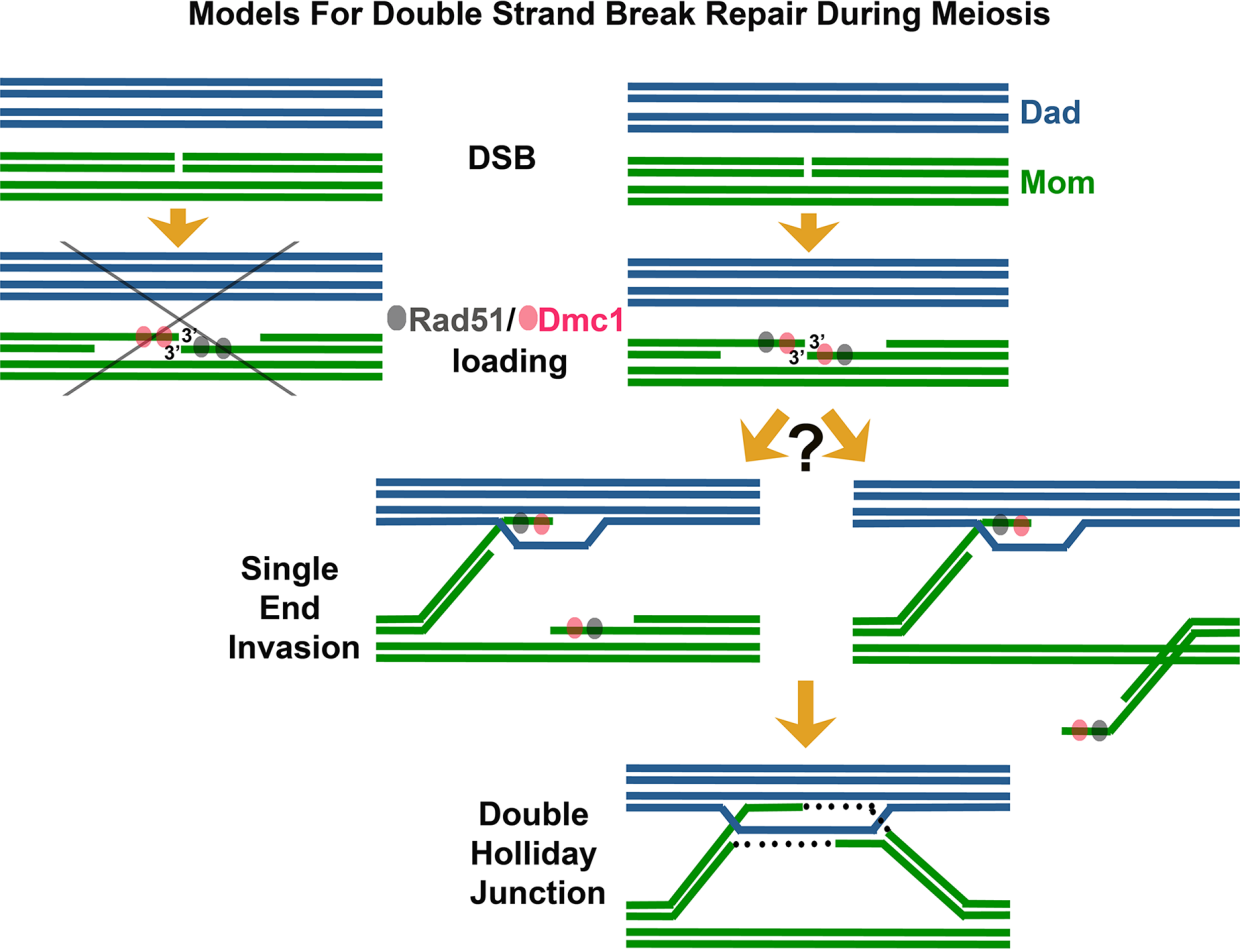
Revising models for meiotic recombination. Illustration depicts steps involved in the repair of meiotic DSBs. A DSB is suffered by one DNA duplex (chromatid) of a replicated parental chromosome (in this case, the “Mom” chromosome, green). 5′ termini on either side of the DSB are resected, and 3′ ssDNA tails assemble with the RecA homologs Rad51 (gray) and Dmc1 (pink) to create nucleoprotein filaments competent for strand invasion and exchange with a homologous duplex DNA. Available templates for repair include the sister (green) or either of two homologous chromatids (blue, “Dad” chromosome). The abbreviated pathway on the far left depicts a model based on the idea that Rad51 and Dmc1 assemble differentially on opposite 3′ termini corresponding to a single DSB. Data presented in Brown et al. [1] suggest instead that opposite 3′ termini corresponding to a DSB often load both Rad51 and Dmc1 (pathway at right). Strand invasion by one 3′ end followed by DNA synthesis can lead to a Holliday Junction structure. It remains to be determined whether opposite ends exhibit asymmetric behavior despite their equivalence in terms of Dmc1 and/or Rad51 loading—for example, whether one 3′ end is selectively released to initiate a search for a homologous duplex DNA (left branch) or alternatively whether both 3′ ends at a DSB exhibit equivalent homology search behavior (right branch).

Among the first enzymes at the scene of a DNA break are RecA-family DNA-dependent ATPase proteins, which assemble on the 3′ single-stranded DNA (ssDNA) termini associated with the DSB [6]. The resulting nucleoprotein filaments have the remarkable capacity to interrogate surrounding double-stranded DNA (dsDNA) and melt homologous duplex DNA through strand invasion and exchange events. Strand exchange involves a local reconfiguration of the DNA duplex, whereby a parental strand is displaced while the invading ssDNA filament interacts, via Watson-Crick base pairing, with its complementary DNA strand. During DSBR [5], strand exchange followed by DNA synthesis can result in two complete DNA duplexes linked to one another by crossed-strand structures called Holliday junctions, which in turn may be processed to form crossover events, wherein corresponding sections of DNA duplexes undergo reciprocal exchange. During meiosis, DSBR typically employs two RecA homologs: Rad51 and the meiosis-specific Dmc1 protein [3,7–9]. Prior studies indicate that while budding yeast Rad51 and Dmc1 can exhibit redundant functions in certain contexts [10–12], normally Rad51 acts as an accessory protein to promote the strand invasion activity of Dmc1 [13]. Interestingly, Rad51 and Dmc1 often form overlapping but slightly offset “co-foci” on meiotic chromatin [14,15]. These Rad51-Dmc1 co-foci, also observed in *Arabidopsis* [16], have been cited as evidence that Rad51 and Dmc1 load differentially on opposite ends of the meiotic DSB (Fig 1) [17,18]. Such a dramatic asymmetry in the biochemical composition of DSB ends could facilitate both the differential and coordinated behavior of those ends during meiotic recombination. For example, one model posits that DSB ends are selectively released from axis sites, where DSBs occur [19,20]. Under this model, the Dmc1 end of a meiotic DSB may be released to initiate a homology search while the Rad51 end remains relatively quiescent at the axis [17,18,21].

In their study reported in this issue, Brown et al. [1] use careful observation and clever engineering of strains with reduced meiotic DSB density to refute the idea that Rad51 and Dmc1 load differentially on opposite ends of meiotic DSBs. Instead, the authors argue that Rad51 and Dmc1 co-occupy the 3′ termini of many (perhaps most) DSBs, and that termini are often in an “ends apart” configuration. Using nearest neighbor analysis in conjunction with simulations of a random distribution, these authors demonstrate that Rad51-Dmc1 co-foci display a pair-wise distribution in which partner co-foci are separated by up to ~400 nm. Furthermore, they examine meiotic nuclei carrying a maximum of two DSB sites and find that the numbers of Rad51 and Dmc1 foci observed are incompatible with a model in which Rad51 and Dmc1 load differentially on opposite ends of DSBs. Finally, the authors present a pioneering use of dSTORM microscopy to analyze the in vivo arrangement of Rad51 and Dmc1 nucleoprotein filaments. Their super-resolution images reveal that Rad51 and Dmc1 assemble short filaments spanning only ~100 nt of ssDNA at meiotic DSB termini. Although perhaps surprising given the capacity of RecA to form long filaments on ssDNA in vitro, the observations resonate well with recent single molecule studies that suggest RecA-mediated strand invasion occurs through discrete capture events involving just eight nucleotides of homology [22].

These observations lead Brown et al. [1] to conclude that short Rad51 and Dmc1 filaments assemble on a single DSB end, with a corresponding partner end situated up to ~400 nm away. These findings appear to put to rest the idea that differential loading of Rad51 and Dmc1 is the basis for an asymmetric behavior of DSB ends during meiotic recombination, at least in budding yeast. The authors in fact propose the alternative idea that the ends of a meiotic DSB are equivalent in their axis release and search behavior.

It remains to be proven whether the behavior of meiotic DSB ends is equivalent during the search for a proper repair partner. The 200–400 nm spacing between Dmc1 and/or Rad51-decorated DSB termini is consistent with the idea that DSB ends are both released from the axis and undergo an equivalent search process but does not rule out the possibility that a single DSB end is released while the other remains at the axis. As discussed in Kim et al. (2010), given a 15 Kb chromatin loop size [19], the corresponding length of a released chromatin arm (6-fold compacted relative to naked DNA) is estimated to be ~350 nm.

It will thus be interesting to know whether ~400 nm-separated Rad51-Dmc1 partner foci localize to separate chromosome axes (a possible expectation if only one end is released) or whether they often show non-axis localization. Visualizing contiguous axes is challenging in early meiotic nuclei of wild-type yeast, but this analysis may be feasible in *zip1* mutants, in which pairs of Dmc1-Rad51 co-foci are apparent at later meiotic stages on aligned and traceable axes [1].

Together, the experiments presented by Brown et al. [1] underscore the tremendous value of in vivo high-resolution observation for building models of dynamic cellular processes. The important discovery that Dmc1 and Rad51 can co-assemble on both ends of a DNA DSB indicates that if pre-invasion DSB termini behave differently, this is due to processes independent of Rad51 and Dmc1 assembly. These data also nicely set the stage for future use of super-resolution microscopy to "capture" the relative arrangement of Rad51 and Dmc1 at individual DSB termini, as well as their spatial arrangement relative to the chromosome axis.
